# Pre-Vaccination Frequencies of Th17 Cells Correlate with Vaccine-Induced T-Cell Responses to Survivin-Derived Peptide Epitopes

**DOI:** 10.1371/journal.pone.0131934

**Published:** 2015-07-15

**Authors:** Tania Køllgaard, Selma Ugurel-Becker, Manja Idorn, Mads Hald Andersen, Jürgen C. Becker, Per thor Straten

**Affiliations:** 1 Center for Cancer Immune Therapy (CCIT), Department of Hematology, Copenhagen University Hospital, Herlev, Denmark; 2 Department of Dermatology, University of Würzburg, Würzburg, Germany; 3 General Dermatology, Medical University of Graz, Graz, Austria; Mie University Graduate School of Medicine, JAPAN

## Abstract

Various subsets of immune regulatory cells are suggested to influence the outcome of therapeutic antigen-specific anti-tumor vaccinations. We performed an exploratory analysis of a possible correlation of pre-vaccination Th17 cells, MDSCs, and Tregs with both vaccination-induced T-cell responses as well as clinical outcome in metastatic melanoma patients vaccinated with survivin-derived peptides. Notably, we observed dysfunctional Th1 and cytotoxic T cells, i.e. down-regulation of the CD3ζchain (p=0.001) and an impaired IFNγ-production (p=0.001) in patients compared to healthy donors, suggesting an altered activity of immune regulatory cells. Moreover, the frequencies of Th17 cells (p=0.03) and Tregs (p=0.02) were elevated as compared to healthy donors. IL-17-secreting CD4^+^ T cells displayed an impact on the immunological and clinical effects of vaccination: Patients characterized by high frequencies of Th17 cells at pre-vaccination were more likely to develop survivin-specific T-cell reactivity post-vaccination (p=0.03). Furthermore, the frequency of Th17 (p=0.09) and Th17/IFNγ^+^ (p=0.19) cells associated with patient survival after vaccination. In summary, our explorative, hypothesis-generating study demonstrated that immune regulatory cells, in particular Th17 cells, play a relevant role for generation of the vaccine-induced anti-tumor immunity in cancer patients, hence warranting further investigation to test for validity as predictive biomarkers.

## Introduction

Immune regulatory cells (e.g. regulatory T cells (Tregs), myeloid derived suppressor cells (MDSC), tumor associated macrophages) have been shown to modulate anti-tumor immunity in cancer patients through various mechanisms, which can result in the suppression of anti-tumor immune responses. More recently, we have demonstrated that these regulatory cells (e.g. factor forkhead box P3 (Foxp3) positive Tregs and tolerogenic dendritic cells) in cancer patients are subject to regulatory cytotoxic T cells themselves [1]. Thus, the outcome of any immune therapeutic intervention, and in particular active immunization by vaccines to treat cancer, are likely to be affected by this complex immune regulatory network. Consequently, current immune therapeutic strategies may be improved by modulating these immune regulatory networks towards stronger anti-tumor immune responses. However, to date our understanding of these complex networks operative both in the tumor micro- and macroenvironment is still rudimentary [2–5].

In the present study, we determined the impact of immune regulatory cells among peripheral blood mononuclear cells (PBMC) on both vaccination-induced T-cell responses and clinical outcome in a subgroup of patients treated in a phase II clinical trial for advanced melanoma. Results from this trial demonstrated that vaccination with survivin-derived peptides in conjunction with Montanide ISA51 induced survivin-specific T-cell responses (SSTR) detectable *ex vivo* in almost one third of the vaccinated patients [6]. Notably, a correlation between the induction of SSTR and clinical outcome was evident: Patients mounting SSTR achieved both a higher disease control rate and a prolonged overall survival (OS) compared to patients with no SSTR [6].

Th17 cells, characterized by a CD4^+^IL-17A^+^ phenotype, have initially been described in immune response to parasites and subsequently in autoimmune diseases and inflammation [7]. However, the relevance of Th17 cells for tumor immunology is still controversial. Indeed both a tumor-promoting as well as a suppressing effect of Th17 cells have been reported [8,9]. In a whole-cell vaccination trial for prostate cancer, pre-vaccination frequencies of Th17 cells, but not Tregs, inversely correlated with time to disease progression [10]. On the other hand, frequencies of Th17 cells increased after immune checkpoint blockade with ipilimumab or tremelimumab, which correlated with improved OS [11].

MDSC are present in increased frequencies in cancer patients compared to healthy donors. After CD14^+^HLA-DR^-^ MDSC were initially reported to be increased in melanoma patients [12], this observation was subsequently expanded to other cancer types such as prostate and renal cell cancer (RCC) [13]. MDSC-mediated suppression of T cells include down-regulation of the CD3ζ chain of the T-cell receptor (TCR) complex and induction of Tregs [14]. Tregs are potent inhibitors of the immune system and suppress both proliferation of and cytokine-production by cytotoxic T cells [15]. Elevated levels of Tregs have been detected both in the tumors and in peripheral blood of cancer patients [16].

Here, we scrutinized the effect of pre-vaccination immune regulatory cells on the immunological and clinical outcome of an anti-tumor vaccination, demonstrating that particularly the frequency of IL-17-secreting CD4^+^ T cells is associated with these endpoints.

## Results

### Stage IV melanoma patients have impaired T-cell reactivity

**Fig 1:**
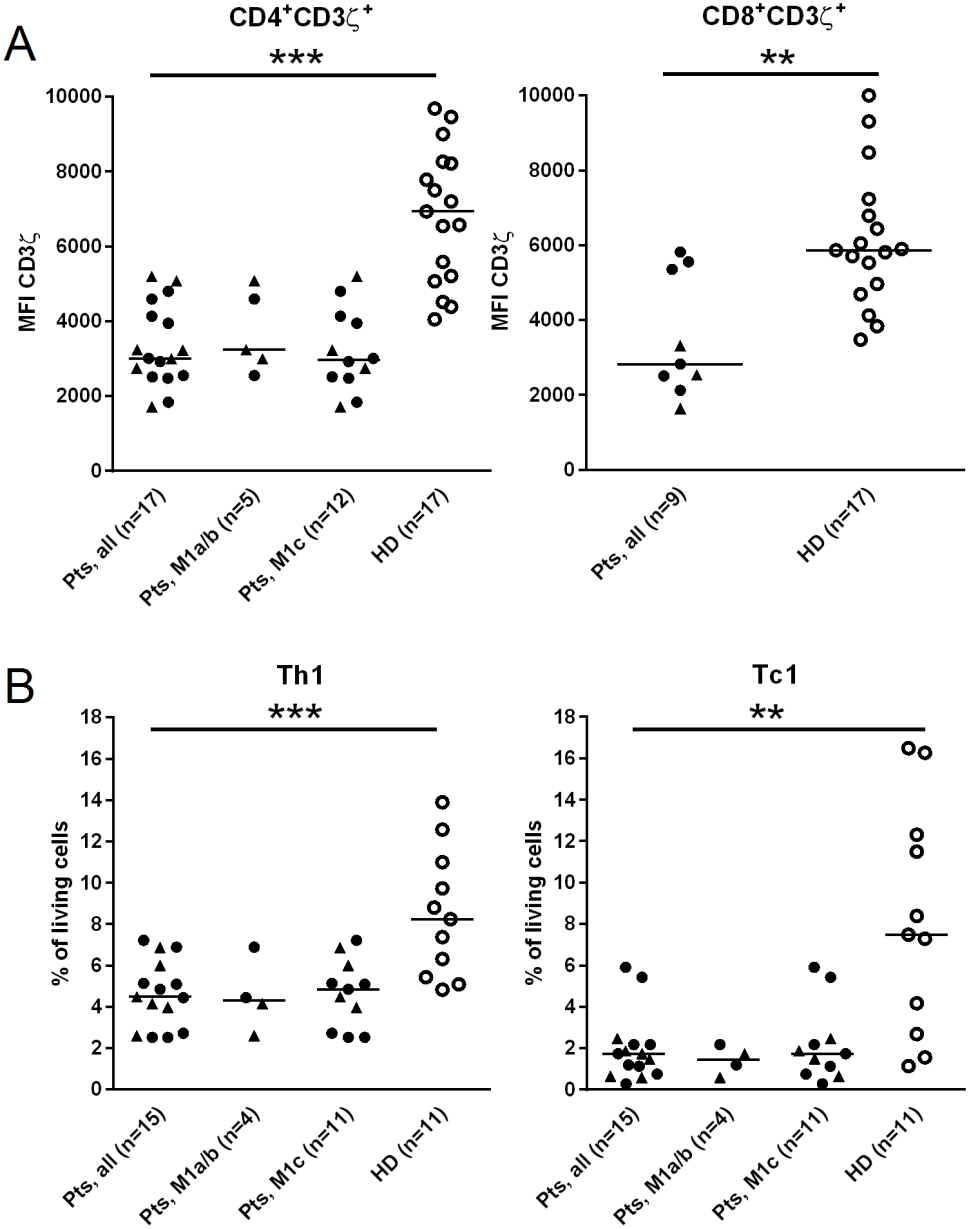
Reduced CD3ζ-expressing and IFNγ-secreting T cells in peripheral blood of stage IV melanoma patients. Peripheral blood mononuclear cell (PBMC) samples obtained from melanoma patients pre-vaccination and from healthy donors (HD) were analysed by flow cytometry using antibodies reacting with CD3ζ chain (A) and IFNγ (B). Cells were stained after stimulation with phorbol myristate acetate (PMA) and ionomycin for four hours. Results are depicted for CD4^+^ (left panel) and CD8^+^ (right panel) T cells. Frequencies of IFNγ-secreting cells were calculated as percentage of living PBMC. Results are given for all patients as well as stratified to M category. Triangles for female and circles for male indicate patient’s gender. Horizontal bars represent median values. MFI, mean fluorescence intensity. *p<0.05, **p<0.01, ***p<0.001.

In order to establish the functional activity of the adaptive cellular immune system in our cohort of stage IV melanoma patients (Table 1), we first analysed circulating T cells for their expression of the CD3ζ chain and their capacity to secrete IFNγ in response to stimulation with PMA and ionomycin. These analyses revealed a reduced expression of the CD3ζchain in both CD4^+^ and CD8^+^ T cells as compared to healthy donors (p<0.0001 and p = 0.001, respectively; Fig 1A, S1 Fig, Table 2). We stratified patients according to gender and M category and found that CD3ζ-chain expression on CD4^+^ T cells did not differ between males and females or between patients with limited (M1a/b) *versus* advanced (M1c) metastatic disease (Fig 1A, left plot). Due to the limited number of patients analysed for CD3ζ-chain expression on CD8^+^ T cells (n = 9), these data were not stratified (Fig 1A, right plot).

**Table 1:** Patient characteristics.

Characteristics		n = 17 (100%)
**Gender**	Male	10 (58.8)
	Female	7 (41.1)
**Median age (years)**	Range	54 (22-79)
**HLA-type**	A1	8 (47.1)
	A2	10 (58.8)
	B35	7 (41.1)
**M stage (AJCC)**	M1a/b	5 (29.4)
	M1c	12 (70.6)
**Survivin-specific T-cellreactivity (SSTR)**	Positive	6 (35.3)
	Negative	8 (47.1)
	Not assessed	3 (17.6)
**Best overall response**	CR	2 (11.8)
	PR	2 (11.8)
	SD	3 (17.6)
	PD	10 (58.8)
**Objective response**	CR + PR	4 (23.5)
**Disease control**	CR + PR + SD	7 (41.1)

**Table 2:** Frequencies of cell subsets in PBMC from melanoma patients.

PBMC subset	Pre-vaccination	Post-vaccination	Healthy donors
**^a^ Th17**	0.5±0.4 (n=15), p=0.03	0.7±0.7 (n=9), *p= 0.01	0.2±0.1 (n=11)
**^a^ Tregs**	3.5±1.4 (n=17), p=0.02	3.5±1.9 (n=10), p=n.s.	2.6±0.6 (n=20)
**^a^ MDSC**	2.1±2.7 (n=17), p=n.s.	2.9±2.0 (n=10), p=0.006	0.8±0.7 (n=11)
**^b^ CD4^+^CD3ζ^+^**	3351±1054 (n=17), p<0.0001	2696±1172 (n=10), p<0.0001	6819±1797 (n=17)
**^b^ CD8^+^CD3ζ^+^**	3519±1520 (n=9), p=0.001	850±1134 (n=10), p=0.0002	6128±1816 (n=17)
**^a^ Th1**	4.6±1.6 (n=15) p=0.0005	5.6±2.8 (n=9), p= 0.08	8.6±3.2 (n=11)
**^a^ Tc1**	2.0±1.6 (n=15), p= 0.001	3.2±3.1 (n=8), p=0.04	7.8±5.7 (n=11)

Th17= CD4^+^IL17^+^, Tregs= CD4^+^CD25^+^CD127^-^, MDSC=CD14^+^HLA-DR^-^,Th1 = CD4^+^IFNγ^+^, Tc1= CD8^+^ IFNγ^+^.

^a^ % of living cells

^b^ MFI

P-values are comparison between cancer patients and healthy donors

Reduced frequencies of CD4^+^IFNγ^+^ T cells (Th1) and CD8^+^IFNγ^+^ T cells (Tc1) were found in melanoma patients at pre-vaccination as compared to healthy donors (p = 0.0005 and p = 0.001, respectively; Fig 1B, Table 2). Th1- and Tc1 frequencies did not differ between males and females or between patients with limited (M1a/b) *versus* extensive (M1c) tumor spread (Fig 1B).

### Increased frequencies of immune regulatory cells in peripheral blood from melanoma patients

**Fig 2:**
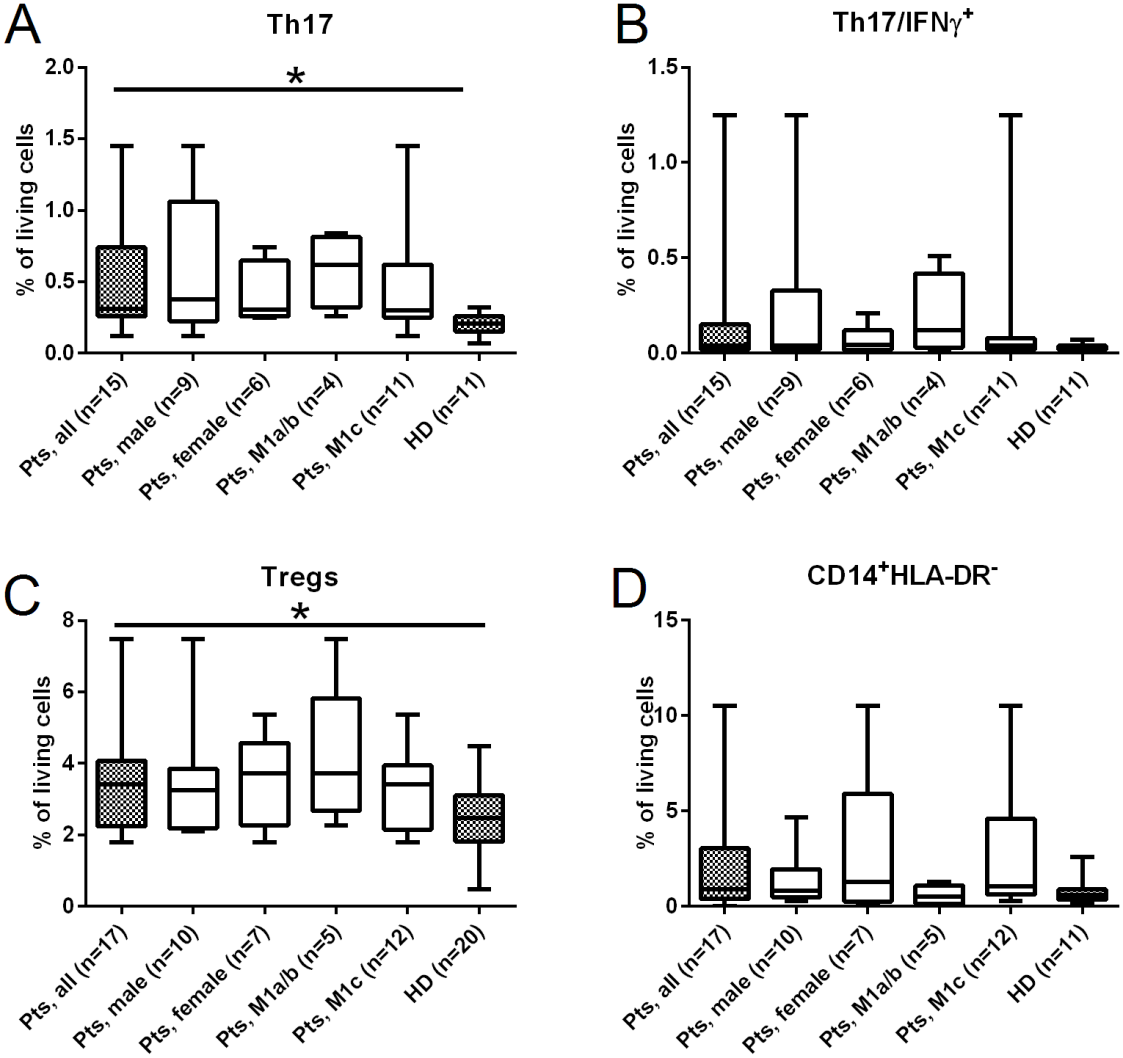
Increased frequencies of immune regulatory cells in peripheral blood of stage IV melanoma patients. PBMC samples obtained from melanoma patients pre-vaccination and from HD were analysed for the presence of Th17 (**A**), IFNγ-secreting Th17 (**B**), Tregs (**C**), and MDSCs (**D**) using flow cytometry with the staining protocol described in the materials and methods section. Frequencies were calculated as percentage of living PBMC. Results are depicted for all patients as well as stratified according to gender and metastatic stage (M category). The horizontal line in the middle of each box indicates the median, whereas the top and bottom borders of the box mark the 75th and 25th percentiles, respectively. The whiskers above and below the box represent the data range. *p<0.05, **p<0.01, ***p<0.001.

Next, we investigated the frequency of the immune regulatory cell subsets present in the peripheral blood of melanoma patients prior to survivin-specific vaccination; for this analysis, we focussed on Th17 cells, Tregs and CD14^+^HLA-DR^-^ MDSC. This approach allowed us not only to determine the frequency of Th17 cells per se, but also to determine the frequency of those Th17 cells able to produce IFNγ after stimulation with PMA and ionomycin for four hours. Due to low cell numbers, frequencies of Th17 cells could be analysed in 15 of 17 patients only. We observed significantly higher frequencies of Th17 cells in melanoma patients as compared to healthy donors (p = 0.03) (Fig 2A, S2 Fig, Table 2). By quantification of Tregs (CD4^+^CD25^+^CD127^-^) in the circulation of melanoma patients, we also observed an increased frequency as compared to healthy donors (p = 0.02) (Fig 2C, S2 Fig, Table 2). Treg frequencies were not associated with the patients’ M category. In addition, no difference was found between males and females. No difference was found between frequencies of IL-17-secreting CD8+ T cells in patients versus healthy donors (data not shown).

We defined MDSC as lineage^neg^, CD14^+^ and HLA-DR^neg^. In addition, these monocytic-like MDSC expressed CD11b and CD33 (S2 Fig). The highest frequencies of CD14^+^HLA-DR^-^ MDSC were observed in melanoma patients compared to healthy donors (p = 0.2). However, this was largely due to profoundly increased MDSC frequencies in 4 patients with high tumor stage (M1c) (i.e. 10.5%, 5.9%, 4.7% and 4.4% (Fig 2D, Table 2). We also investigated the frequency of CD15^+^ polymorphonuclear MDSC in blood from patients. For CD15^+^HLA-DR^-^ polymorphonuclear MDSC no differences were found between patients and healthy donors (data not shown).

### Th17-cell frequencies in pre-vaccination blood samples correlate with vaccine-induced T-cell responses

**Fig 3:**
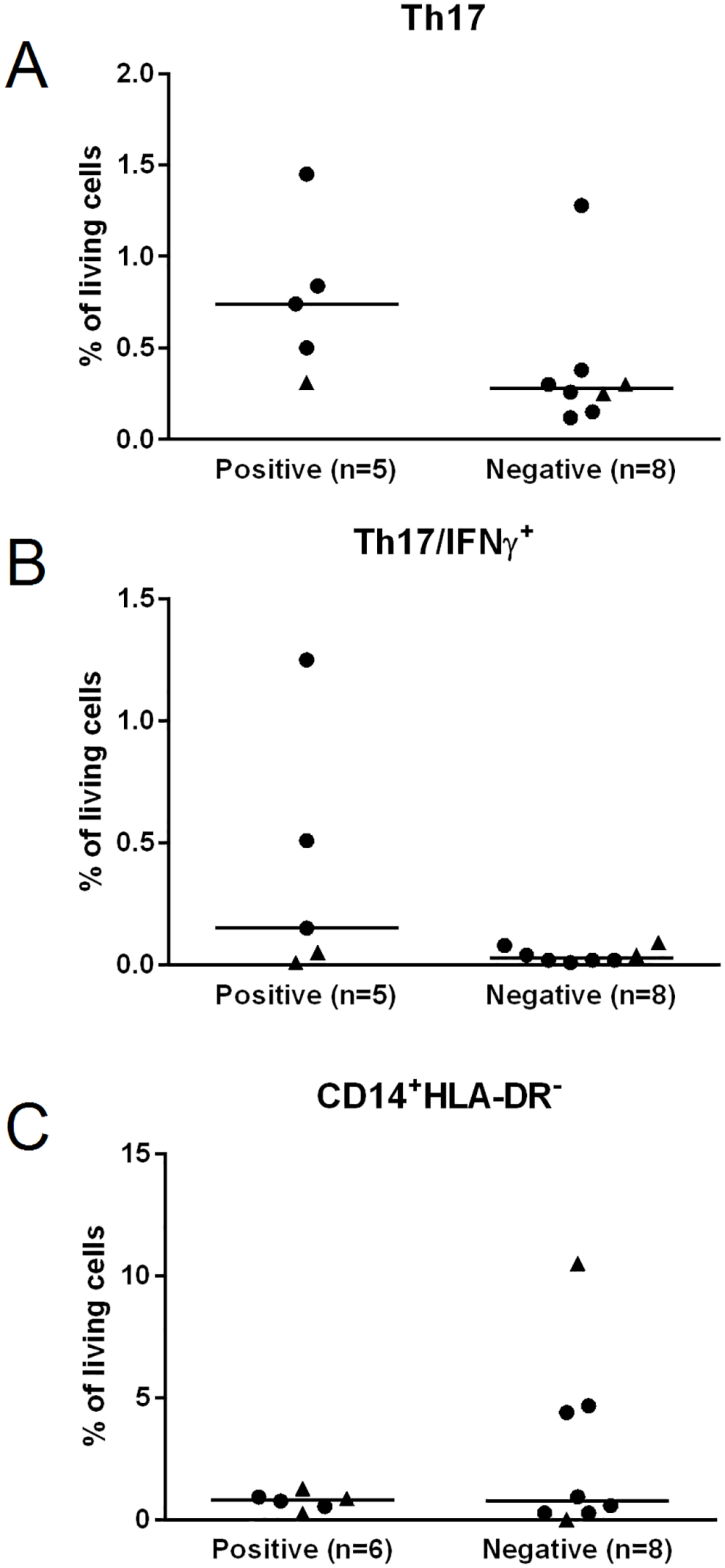
Frequencies of immune regulatory cells pre-vaccination correlate with vaccine-induced T-cell responses. Frequencies of Th17 (**A)**, IFNγ-secreting Th17 cells (**B)**, and MDSC **(C)** depicted as percentage of living cells were grouped according to whether they mounted survivin-specific T-cell responses (SSTR) or not. Patient’s gender is indicated by triangles for female and circles for male. *p<0.05, **p<0.01, ***p<0.001.

The main aim of our study was to generate an initial line of evidence if the frequency of regulatory immune cells may serve as a predictive biomarker for the response to anti-tumor vaccine therapy. Consequently, we investigated a possible association of the extent of vaccine-induced immune responses, namely SSTRs, and the frequencies of Th17 cells, Tregs and MDSC. These analyses revealed that vaccine-induced SSTR associated with increased frequencies of Th17 cells at pre-vaccination (p = 0.03; Fig 3A). Akin, patients with higher frequencies of IFNγ-secreting Th17 cells pre-vaccination were more likely to develop vaccine-induced SSTR after vaccination (p = 0.14; Fig 3B). High frequencies of MDSC in pre-vaccination blood seemed to predict a low chance of developing SSTR upon vaccination. Indeed, none of three patients with the highest levels of MDSC at pre-vaccination showed any SSTR at post-vaccination (Fig 3C); unfortunately, the fourth patient could not be evaluated for vaccine-induced SSTR due to low numbers of PBMC that could be isolated from the patient’s blood. Treg frequencies did not correlate with SSTR (data not shown).

### Pre-vaccination frequencies of Th17 cells in PBMC associated with better survival

**Fig 4:**
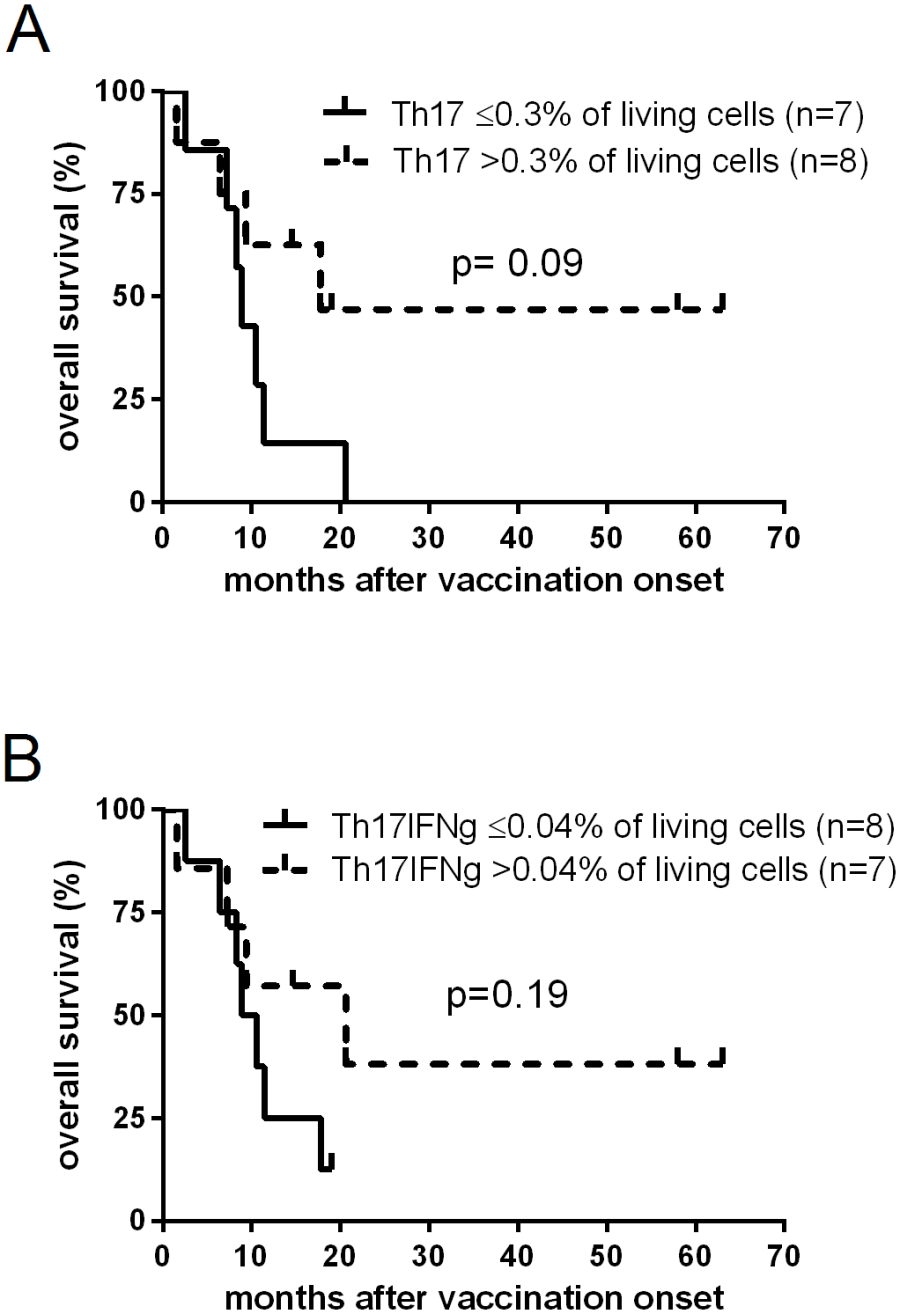
Pre-vaccination frequencies of Th17 cells and survival. Kaplan-Meier plots depicting the probability of overall survival of melanoma patients vaccinated with survivin-derived peptides according to pre-vaccination frequencies of Th17 cells (**A**), and IFNγ-secreting Th17 cells (**B**); groups were separated by Th17 ≤0.3% (solid line) or >0.3% (dotted line) as well as IFNγ-secreting Th17 ≤0.04% (solid line) or >0.04% (dotted line) of living cells, respectively. Statistical differences between groups were calculated using the log rank test; censored observations are indicated by vertical bars.

Based on our previous findings demonstrating that vaccine-induced SSTR correlate with improved disease control resulting in better OS [6], we hypothesized that pre-vaccination frequencies of regulatory immune cells may also correlate with OS. Albeit only a trend, those patients with high pre-vaccination frequencies of Th17 or IFNγ-secreting Th17 cells had a better OS and median survival compared to patients with lower frequencies of these cell subsets (p = 0.09 and p = 0.19, respectively; Fig 4A and 4B). Neither pre-vaccination Tregs nor CD14^+^HLA-DR^-^ MDSC frequencies correlated significantly with patient survival.

## Discussion

The advent of clinically effective immune therapies, i.e. immune checkpoint blocking antibodies, exerting impressive clinical responses in a substantial subset of melanoma patients, urged the demand for predictive biomarkers for immune therapy, since these impressive responses are associated with potentially hazardous side effects [17]. The immunological and thus clinical effect of any immune therapeutic intervention and in particular active immunization are likely to be affected by immune regulatory networks. Thus, detailed analyses of these regulatory networks are likely to allow identification of not only predictive biomarkers, but also possible means to improve current immune therapeutic strategies. However, scrutinizing these immune regulating networks operative both in the tumor micro- and macroenvironment is hampered by the fact that adequate biomaterial has to be collected in a way which allows such analyses—a task which has only been approached in a limited number of clinical trials [18,19]. In order to add to the still limited knowledge, we performed an exploratory analysis of a possible correlation between pre-vaccination Th17 cells, Tregs and MDSCs in peripheral blood with both vaccination induced T-cell responses as well as clinical outcome in metastatic melanoma patients vaccinated with survivin-derived peptides.

The first set of analyses demonstrated a generally impaired function of the adaptive immune system: The expression of the CD3ζ chain on CD4^+^ and CD8^+^ T cells as well as the frequency of IFNγ-secreting CD4^+^ and CD8^+^ T cells in response to stimulation with PMA and ionomycin in stage IV melanoma patients were significantly reduced compared to healthy donors suggesting a state of immune exhaustion, which is well established in settings of continued antigen-stimulation of T cells such as chronic infections, autoimmune diseases and advanced cancer [20]. Notably, this immune exhaustion is reversible: In breast cancer patients CD3ζ expression normalizes after surgical tumor resection [20]. Thus, presence of the tumor is causatively associated with down-regulation of CD3ζ. Our data, however, clearly demonstrate that this state of immune exhaustion is not associated with tumor burden only, as it was also observed in patients with decreased tumor stage. Thus, this would point to down-regulation of CD3ζ as an early event in tumor development as has been suggested before [20,21].

Determining the frequency of immune regulatory cells, i.e. Th17 cells, Tregs and MDSC, in peripheral blood prior to vaccination, we confirmed previous reports that these cell subsets are elevated in advanced cancer patients [22–24]. However, in our cohort of melanoma patients the increased frequencies of CD14^+^HLA-DR^-^ MDSCs were correlated with metastatic stage, whereas Th17 cells and Treg frequencies were not. To this end, it was previously reported that increased frequencies of MDSCs, defined as CD11^+^CD33^+^HLA-DR^-^ cells, correlated significantly with tumor burden in breast cancer patients [25]. The functional immune suppressive function of MDSC in melanoma has been recently evidenced by the correlation of increased levels of MDSCs with the absence of melanoma-antigen-specific T cells [26]. Thus, we tested whether pre-vaccination frequencies of Th17 cells, Tregs or MDSC may serve as predictive biomarkers for the immunological and clinical effect of anti-tumor vaccines. For this purpose, we stratified the patients into two groups, i.e. patients mounting a SSTR or not. This analysis revealed that the highest frequencies of CD14^+^HLA-DR^-^ MDSC were present in patients not mounting a SSTR. Notably, it has been demonstrated before in a different setting that MDSC impair anti-tumor vaccinations [27]. In addition, it was recently demonstrated that MDSC frequencies predicted survival of stage IIIc-IV melanoma patients independently of the AJCC-M category [28]. Thus, MDSC-depleting strategies could potentially improve the outcome of anti-tumor vaccines [29].

Herein, we demonstrate that increased frequencies of Th17 cells at pre-vaccination characterize patients mounting a SSTR after vaccination. In addition, it has been shown that IFNγ^+^-secreting IL-17^+^ CD4^+^ T cells are increased in inflamed tissues and, importantly, have been reported to mediate tumor regression in a mouse model via an IFNγ- dependent mechanism [30,31]. In the present study, we found that patients with higher frequencies of IFNγ-secreting Th17 cells were more likely to develop SSTR. Still, the prevalence of Th17 cells in tumors and their impact on tumor immunology *in vivo* remains controversial, and both anti- and pro-tumorigenic roles have been described [24,32]. To this end, the role of Th17 cells in tumor immunity depend upon several factors such as tumor tissue, cytokine signals and interplay with other immune cells. Our findings corroborate with anti-tumor functions of Th17 cells by facilitating the induction of anti-tumor T-cell responses by vaccination.

Since Th17 and MDSC frequencies were associated with vaccine-induced SSTR, and vaccine-induced SSTR were correlated with clinical outcome, it was not surprising that pre-vaccination frequencies of Th17 cells and MDSC showed at least a trend towards a prediction of clinical outcome. Albeit only a trend, which may be due to the low number of patients analysed, patients with higher frequencies of Th17 and IFNγ-secreting Th17 cells experienced a higher survival rate compared to patients with lower frequencies of these cells. Patients with high MDSC frequencies at pre-vaccination were characterized by an impaired survival; however, since MDSC levels were also correlated with the extent of metastatic spread, these patients had a poorer prognosis anyhow.

In summary, our results from this explorative, hypothesis-generating study suggest that pre-vaccination frequencies of immune regulatory cells, in particular Th17 cells, correspond with vaccine-induced T-cell responses. Indeed, further characterization of circulating immune regulatory cells in clinical trials testing immunotherapy are warranted and may not only deliver predictive biomarkers but also clues how to improve immune therapy.

## Materials and Methods

### Patients and clinical samples

PBMC from 17 melanoma patients treated in a single-institution, prospective phase-II vaccination trial for stage IV melanoma (ClinicalTrials.gov ID: NCT00108875) [6], were obtained immediately prior to the first vaccination (pre-vaccination). PBMC were cryopreserved until further analysis. Per protocol, patients had to have disease progression after at least one previous systemic therapy (dacarbazine (DTIC)), distant metastases not amendable by surgery, and a HLA type of A1, A2 and/or B35. Patients were allocated to M categories according to the 2009 AJCC classification [33]: M1a/b, metastases to the skin, subcutis, lymph nodes or lung; M1c, metastases to other organs. Detailed patient characteristics are presented in Table 1. The extensive trial eligibility criteria and vaccination regimens and doses were described previously in Becker et al [6]. Briefly, patients received HLA-matched peptides derived from the anti-apoptotic protein survivin emulsified in Montanide by subcutaneous injection. This study was approved by the local Ethics Committee (Ethics Committee of the Medical Faculty of the University of Würzburg; No 07/2003). From all patients written informed consent was obtained before onset of any study procedures. For the present analysis, best overall response (complete response (CR), partial response (PR), stable disease (SD), or progressive disease (PD) according to RECIST 1.0 [34]) was grouped into response, i.e. progression arrest (CR/PR/SD), and non-response (PD). Cryopreserved PBMC from healthy donors were used as controls; to match the mean age of the study patients (54 years, range 22–79), we used 11 healthy donors from the laboratory staff (mean age 63 years, range 61–68) as well as 20 healthy donors from the blood bank at Rigshospitalet (mean age 38 years, range 17–67), Copenhagen, Denmark.

### Flow cytometry analyses of PBMC subsets

CD3ζ-chain expression by T cells was analysed by using intracellular staining (ICS) and flow cytometry according to the following protocol: First, cryopreserved PBMC were thawed, washed and placed on ice in 100 μL PBS buffer supplemented with 2% FCS. Mouse serum (TriChem APS, Skanderborg, Denmark) was added for 10 min to block nonspecific binding in all stainings carried out. Next, cells were stained with the following monoclonal antibodies (mAbs) anti-CD3-PE-Cy7 (SP34-2, BD, Albertslund, Denmark), anti-CD4-PerCP (L200, BD), anti-CD8-PacificBlue (DK25, Dako Denmark A/S, Glostrup, Denmark) as well as LIVE/DEAD Fixable Near-IR Dead Cell Stain (Invitrogen, life technologies, Naerum, Denmark) to exclude dead cells. After staining for 30 minutes on ice cells were fixed and permeabilized overnight with Fixation/Permeabilization and Permeabilization Wash Buffer (eBioscience, Aarhus, Denmark). The next day, cells were washed and stained with anti-CD3ζ (CD247)-FITC (G3, ThermoScientific, Hvidovre, Denmark) for 20 min on ice. After two final washes cells were ready for analyses by flow cytometry.

For analyses of Th17, Tc17, Th1 and Tc1 cells, cryopreserved PBMC were thawed and rested overnight in X-vivo15 (BioWhittaker, Verviers, Belgium). The following day, 2 μL of Leukocyte Activation Cocktail with GolgiPlug (LAC) (containing phorbol myristate acetate (PMA), ionomycin and brefaldin A) (BD) was added to the PBMC and incubated for four hours at 37°C/5%CO_2_. As negative control, 2 μL of GolgiPlug was added to the PBMC. Cells were stained with anti-CD4-PacificBlue (RPA-T4, Biolegend, London, UK), anti-CD3-PE-Cy7 (BD), anti-CD8-FITC (RPA-T8, BD) and LIVE/DEADFixable Near-IR Dead Cell Stain (Invitrogen). After fixation and permeabilization cells were stained with anti-IFNγ-PE (4S.B3, eBioscience) and anti-IL-17A-PerCP-Cy5.5 (eBio64DEC17, eBioscience) and analysed by flow cytometry.

PBMC were thawed and analysed for frequencies of Tregs and CD14^+^HLA-DR^-^ MDSC. The mAbs used for Tregs were anti-CD127-FITC (eBioRDR5, eBioscience), CD25-APC (eBioscience), anti-CD3-APC-Cy7 (SK7, BD), anti-CD4-PerCP (BD), and anti-CD8-PacificBlue (Dako). The mAbs panel for detection of CD14^+^HLA-DR^-^ MDSC consisted of anti-CD14-APC-Cy7 (MφP9, BD), anti-CD15-PacificBlue (SSEA-1, Biolegend), anti-HLA-DR-PerCP (L243, BD), and anti-CD11b-APC (ICRF44, eBioscience). Furthermore, anti-CD3-PE-Cy7 (BD), anti-CD56-PE-Cy7 (B159, BD), and anti-CD19-PE-Cy7 (SJ25C1, BD) were used as lineage markers. CD14^+^HLA-DR^-^ cells were identified within the lineage negative cell population. The LIVE/DEAD-Fixable-Aqua-Dead-Cell—Stain-AmCyan (Invitrogen) was used to exclude dead cells in both the Tregs and CD14^+^HLA-DR^-^ cell analyses.

All cells were analysed using a BD FACSCanto II flow cytometer and BD FacsDiva Software. At least 5x10^5^ cells were acquired and used for analyses. Relevant isotypes were used as controls for all mAbs. To avoid bias, flow analyses of patient and healthy donor PBMC were performed at the same time.

### Survivin-specific T-cell reactivity (SSTR)

Enzyme-linked immunospot (ELISPOT) assays were used to quantify IFNγ-releasing survivin-specific T cells in PBMC samples as described previously [6,35]. Nitrocellulose-bottomed 96-well plates (MultiScreen MAIP N45, Millipore, Schwalbach, Germany) were coated with an anti-IFNγ antibody (1-D1K, Mabtech, Stockholm, Sweden), and nonspecific binding was blocked using AIM-V (Life Technologies, Gaithersburg, MD). Lymphocytes were isolated from heparinized peripheral blood samples of study patients and subsequently incubated overnight at 37°C at different cell concentrations together with T2 cells loaded with HLA-matched survivin epitope-specific peptides used for vaccination. After two washing procedures, the biotinylated detection antibody (7-B6–1-Biotin, Mabtech) was added. Specific binding was visualized using alkaline phosphatase-avidin together with substrate (Life Technologies). The resulting spots were quantified using the AlphaImager System (Alpha Innotech, San Leandro, CA). IFNγ- secretion was considered as a specific response, if the spot count for any of the specific peptides was more than three times the count of background spots (no peptide) in at least two independent experiments. Patients with a detection of a vaccine-induced SSTR at at least one time point (i.e. week 8 or 16 after onset of vaccination) were defined as positive.

### Statistics

Statistical analyses were performed using GraphPad Prism 6.0 (GraphPad Software, CA, USA). Differences between groups were compared using the Mann-Whitney test. Kaplan-Meier plots were performed for survival analyses and differences between groups were tested with the log rank test. p<0.05 were considered significant.

## Supporting Information

S1 Fig Patients showed decreased expression of the CD3ζ chain on T cells. Flourescence histograms representing CD3ζ MFI of gated CD4^+^ (left plot) and CD8^+^ (right plot) T cells from a representative patient and a normal donor. Dark grey = patient, light grey = normal donor.
(TIF)

S2 Fig Gating strategy for analyses of Th17 cells, Tregs and CD14^+^ MDSC. Living single cell PBMC were gated using FSC-A/FSC-H and DCM/FSC-A (first row). Th17 cells were identified as CD3^+^CD8^-^CD4^+^IL-17A^+^ cells and IFNγ Th17 cells were identified as CD3^+^CD8^-^CD4^+^IL-17A^+^ IFNγ^+^ (second row) Tregs were identified as CD3^+^CD4^+^CD25^hi^, CD127^low^ (third row) and MDCS were identified as lineage^-^HLA-DR^-^CD14^+^ (fourth row) In addition, MDSC expressed both CD33 and CD11b. DCM = dead cell marker.
(TIF)
