# Supplementary figures and images for: Pre-Vaccination Frequencies of Th17 Cells Correlate with Vaccine-Induced T-Cell Responses to Survivin-Derived Peptide Epitopes
Source: PLoS One. 2015 Jul 15;10(7):e0131934. doi: 10.1371/journal.pone.0131934 (PMC4503613; doi:10.1371/journal.pone.0131934)

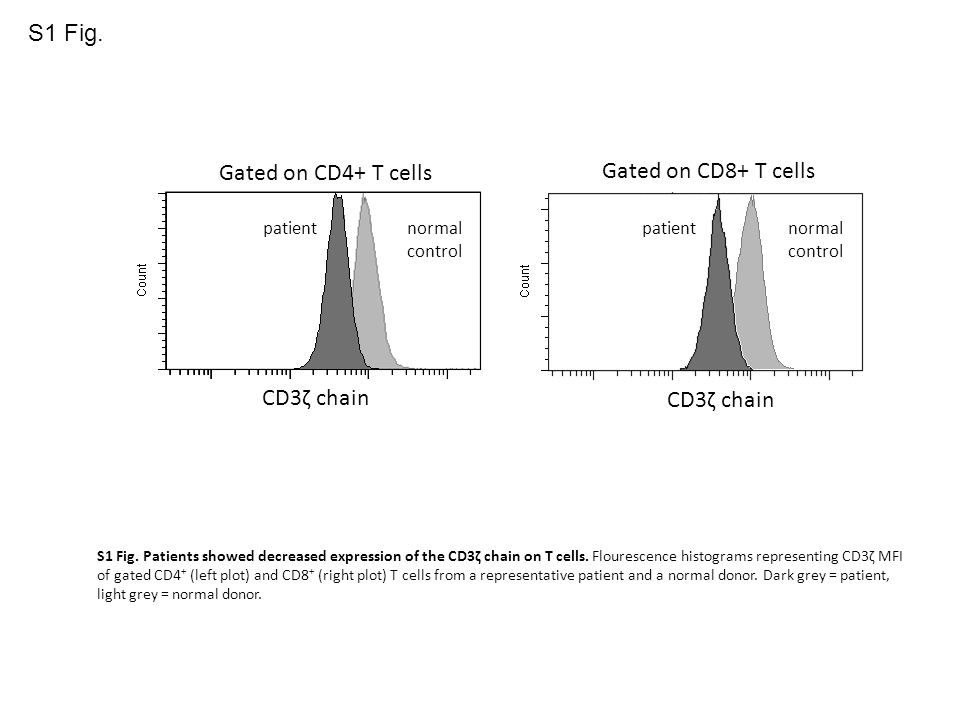

Supplement: S1 Fig — Flourescence histograms representing CD3ζ MFI of gated CD4+ (left plot) and CD8+ (right plot) T cells from a representative patient and a normal donor. Dark grey = patient, light grey = normal donor. (TIF) [file pone.0131934.s001.tif]

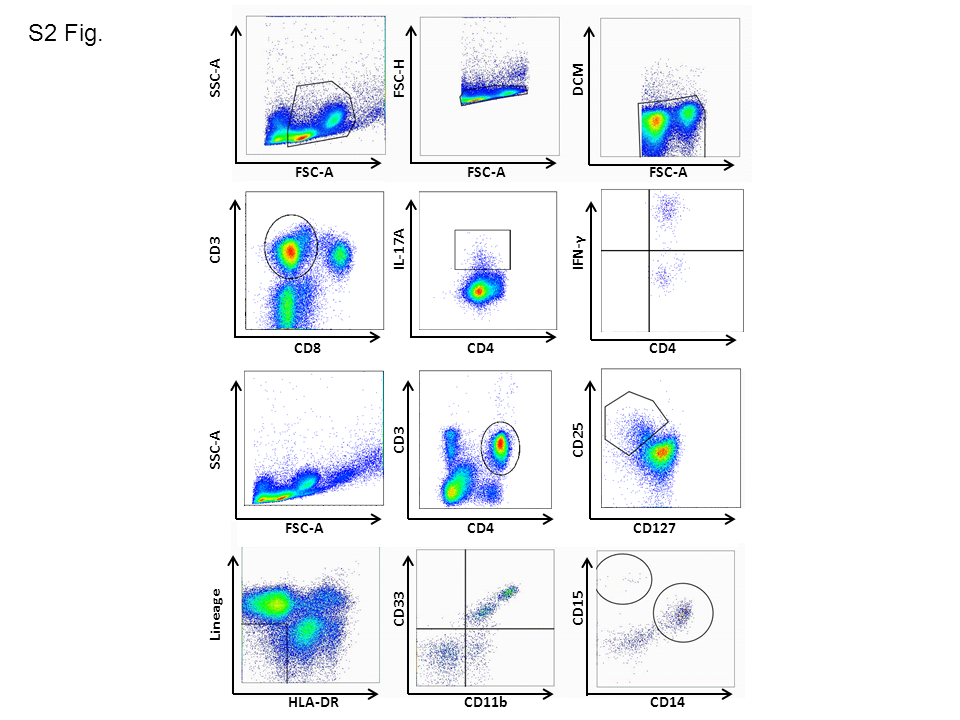

Supplement: S2 Fig — Living single cell PBMC were gated using FSC-A/FSC-H and DCM/FSC-A (first row). Th17 cells were identified as CD3+CD8-CD4+IL-17A+ cells and IFNγ Th17 cells were identified as CD3+CD8-CD4+IL-17A+ IFNγ+ (second row) Tregs were identified as CD3+CD4+CD25hi, CD127low (third row) and MDCS were identified as lineage-HLA-DR-CD14+ (fourth row) In addition, MDSC expressed both CD33 and CD11b. DCM = dead cell marker. (TIF) [file pone.0131934.s002.tif]
